# Optically-Controlled Terahertz Multifunctional Polarization Conversion Metasurface with Reflection and Transmission Modes

**DOI:** 10.3390/mi13091387

**Published:** 2022-08-25

**Authors:** Ying Tian, Lichang Han, Li Yan, Jiayun Wang, Binzhen Zhang, Zan Jiao

**Affiliations:** 1 Sichuan Aerospace Liaoyuan Science and Technology Co., Ltd., Chengdu 610100, China; 2 Key Laboratory of Instrumentation Science & Dynamic Measurement, North University of China, Taiyuan 030051, China

**Keywords:** terahertz, metasurface, multifunction, polarization conversion

## Abstract

In the terahertz band, how integrating multiple functions into a device with a tiny unit structure is a challenge. In this paper, an optically-controlled multifunctional linear polarization conversion metasurface working in the terahertz band is proposed. The reflection and transmission polarization conversion functions can be realized by irradiating the metasurface with pump light with different wavelengths. The metasurface is designed with a multilayer structure, and a photosensitive semiconductor alone is used to control multiple functions, which makes the manipulation of multifunctional devices easy. When the photosensitive semiconductor germanium (Ge) and silicon (Si) are in different states, the metasurface can realize broadband reflection and transmission polarization conversion functions, the corresponding relative bandwidth are 102.4% and 98.9%, respectively, and the work efficiency can be regulated by pump light with different intensity and wavelength. In addition, the working principle of the metasurface is analyzed by eigenmode theory and surface current distributions. The stability of the metasurface to structural parameters and incident angles are discussed.

## 1. Introduction

As a basic feature of polarized electromagnetic waves, it is very meaningful to reasonably control the polarization direction of electromagnetic waves for the application process of electromagnetic waves [1]. The metasurface is a novel two-dimensional synthetic material, usually an ultra-thin surface with sub-wavelength unit cells arranged periodically or nonperiodically. It has the excellent characteristic that the material properties can be controlled by artificial design, and the advantages of thinner size and lighter weight. According to the artificial and reasonable design, the metasurface can effectively control electromagnetic waves. Researchers have successively designed many devices based on metasurfaces, such as holographic imaging [2], vortex beam [3], sensors [4,5], absorbers [6,7,8], and polarization converters [9,10,11,12,13,14,15,16]. A metasurface polarization converter is a device that controls the polarization state of the incident electromagnetic wave with the response between the electromagnetic wave and the resonator. Following recent research, metasurface polarization converters have covered the functions of linear polarization to linear polarization [9], linear polarization to circular polarization [10], and circular polarization to circular polarization conversion [11,12], and its working modes also include reflective [13,14,15] and transmissive [16,17].

At present, the demand for practical application is severely increasing, and the above-mentioned traditional polarization conversion devices without adjustable functions can hardly meet some special occasions. Therefore, researchers embed devices or materials with switchable or controllable functions into the metasurface to realize the controllable function of the metasurface. The most commonly used materials are electronic control devices, such as PIN diode [18], varactor diode [19] and graphene [20,21]; temperature-controlled vanadium dioxide [22,23] and optically-controlled semiconductor [24,25], etc. The electrical properties of these materials or devices can be effectively controlled by corresponding external excitation means to produce different responses to electromagnetic waves. For example, Quader et al. designed a terahertz linear polarization to circular polarization metasurface as a resonator, and the performance of polarization conversion can be controlled by the chemical potential of graphene [20]. Zhang et al. designed a broadband tunable terahertz wave absorber using a double-layer vanadium dioxide square ring array, and the absorption amplitude of the absorber can be controlled by temperature [23]. Zhao et al. designed a broadband linear polarization converter based on photosensitive semiconductor Si, and the polarization conversion efficiency can be controlled by adjusting the intensity of the pump light [24]. These tunable metasurfaces have only one operating mode, which is regulated by external excitation. In recent years, the polarization conversion metasurface with multiple functions has attracted the attention of most researchers [26,27,28,29,30,31,32]. Such metasurfaces tend to have more operating modes and controllable properties than tunable metasurfaces. For example, Yang et al. proposed a reconfigurable polarization conversion metasurface based on a PIN diode, which can realize reflective broadband linear to circular polarization conversion and dual-band linear polarization to linear polarization conversion when the diodes are off or on state [29]. Li et al. designed a metasurface with dual functions of polarization conversion and filtering by embedding vanadium dioxide blocks in the metal resonator, and the two functions can be switched by changing the conductance state of vanadium dioxide [30]. Yang et al. designed a multifunctional device by embedding graphene and photosensitive semiconductor silicon in the metasurface, which can realize wave absorption, linear polarization conversion, and circular polarization conversion by changing the chemical potential of graphene and the conductivity of photosensitive Si [31]. However, the above-mentioned multifunctional metasurfaces can only work in reflective or transmissive modes. There are few reports on polarization conversion metasurfaces that can work in both reflective and transmissive modes. The multifunctional metasurface proposed by Zhao et al. can realize the reflective and transmissive polarization conversion working modes [33]. At the same time, its structure adopts graphene and vanadium dioxide as the control means. It needs two external stimuli, voltage and temperature, to drive the metasurface, so the control mode is complicated. To address this challenge, we adopt a multilayer metasurface embedded photosensitive semiconductor to control the operating modes of reflection and transmission only by pump light.

In this paper, a simple optically-controlled multifunctional polarization conversion metasurface is designed by embedding a photosensitive semiconductor in the resonator. It can be switched between reflective and transmissive modes only by using pump light with different wavelengths, and the working efficiency can be tuned via the intensity of the pump light. When the designed metasurface is excited by pump light with a wavelength of 1550 nm, it works in reflective polarization conversion mode. When the pump light is switched to the wavelength of 800 nm, the working mode of the metasurface is translated to the transmission polarization conversion mode. The polarization conversion efficiency of the two working modes can be tuned by the intensity of the pump light, and the working frequency bands of the two working modes are almost the same.

## 2. Design and Methods

**Figure 1:**
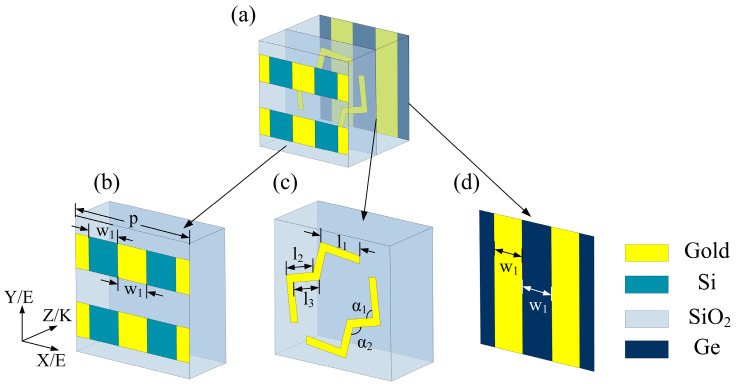
Schematics diagrams of the unit cells of the proposed multifunctional polarization metasurface. (**a**) the top layer, (**b**,**c**) the middle layer, and (**d**) the bottom layer.

The unit structure of the designed optically-controlled multifunctional polarization conversion metasurface is shown in Figure 1, and the pattern layer of the structure is mainly divided into three parts: top layer, middle layer, and bottom layer. The top layer is attached to the SiO_2_ layer of the dielectric substrate with a thickness of 40 μm, and the resonator is a horizontal metal wire in which a square photosensitive semiconductor Si block is embedded, as shown in Figure 1b. The middle layer is a double W-shaped resonator, which is attached to the SiO_2_ layer of the dielectric substrate with a thickness of 50 μm, as shown in Figure 1c. The backplane is formed by arranging the bottom metal lines and photosensitive semiconductors alternately, as shown in Figure 1d. The metal material in the structure is gold, the conductivity is 4.56 × 10^7^ S/m, and the dielectric constant of SiO_2_ is 3.9. Other optimized parameters are as follows: *p* = 100 μm, *w*_1_ = 25 μm, *l*_1_ = 34.4 μm, *l*_2_ = 24.5 μm, *l*_3_ = 22.8 μm, *α*_1_ = 120°, *α*_2_ = 120°.

In the electromagnetic simulation, we perform modeling and simulation through electromagnetic simulation software CST Studio Suite based on the finite integration technique (FIT). The unit cell boundary condition is set along the x-axis and y-axis directions, and the open (add space) boundary condition is set along the z-axis. At this time, the polarization direction of the electric field of the incident electromagnetic wave is perpendicular to the incident surface, and it is polarized along the y-axis direction and incident on the metasurface, as shown in Figure 1b. The polarization conversion performance of the device can be measured by the polarization conversion rate (PCR). The polarization conversion ratio PCR_r_ to reflection mode can be expressed as: PCR_r_ = |r_xy_|^2^/(|r_yy_|^2^ + |r_xy_|^2^ + |t_yy_|^2^ + |t_xy_|^2^), where r_xy_ and r_xy_ are coplanar polarization reflection coefficient and cross-polarization reflection coefficient, and t_yy_ and t_xy_ are coplanar polarization transmission coefficient and cross-polarization transmission coefficient, respectively; Similarly, the polarization conversion rate PCR_t_ for the transmission mode is: PCR_t_ = |t_xy_|^2^/(|r_yy_|^2^ + |r_xy_|^2^ + |t_yy_|^2^ + |t_xy_|^2^).

Photosensitive semiconductors Si and Ge are sensitive to pump light intensity, and their conductivity can be controlled by the pump light intensity of the corresponding wavelength. At room temperature, photosensitive Si can be excited by pump light with a wavelength of less than 1100 nm, while Ge can be excited by pump light with a wavelength of less than 1600 nm [34,35]. According to the fitting of experimental parameters, the relationship between the conductivity of photosensitive Si and the pump light intensity is [36]: σ_Si_ = 4.863 × 10^−4^ × I^2^ + 0.1856 × I + 1.569, which shows that when the pump light intensity increases from zero to 615.5 μJ/cm^2^, the conductivity of photosensitive Si will increase from 0 S/m to 3 × 10^5^ S/m. Furthermore, according to the reference [36], it can be considered that the conductivity of photosensitive Si and Ge is basically the same as the pump light intensity, so the performance of the designed metasurface can be controlled by the pump light intensity of different wavelengths.

In addition, the conceivable production plan of the proposed metasurface is stated here. First, the dielectric substrates are prepared on polysilicon by spin coating technology; second, gold films are prepared by magnetron sputtering on the surface of the corresponding dielectric substrates, and the photosensitive semiconductor Si and Ge are sputtered at the top and bottom layers, respectively; finally, the photosensitive semiconductor and gold are successively etched into patterns, and the two pattern-dielectric layers are adhered to obtain the proposed metasurface.

## 3. Results and Discussion

### 3.1. Optically-Controlled Multiple Operating Modes

**Figure 2:**
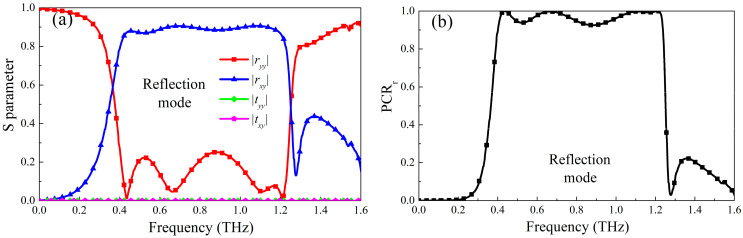
The Simulated results of reflection operating mode (**a**) S parameter; (**b**) PCR_r_.

**Figure 3:**
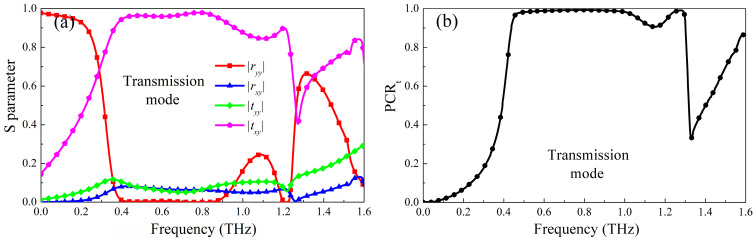
The Simulated results of reflection operating mode (**a**) S parameter; (**b**) PCR_t_.

The results of the simulated reflection and transmission modes are shown in Figure 2 and Figure 3. It can be seen from Figure 2 that when the conductivity of photosensitive Ge is 300,000 S/m, it is in a metallic state and photosensitive Ge is in an insulating state, the designed metasurface works in a reflective polarization conversion mode, the coplanar polarization reflection coefficient in the range of 0.40 THz–1.24 THz is less than 0.3, and the cross-polarization reflection coefficient is greater than 0.8. Because photosensitive Ge is in a metallic state, The bottom layer of the structure is equivalent to a metal grounding plate, which can effectively block the transmission of electromagnetic waves, so the transmission coefficient is almost zero, as shown in Figure 2a. Therefore, according to the formula of PCR, the PCR_r_ in this frequency band is over 90%, and the relative bandwidth is 102.4%, as shown in Figure 2b. However, when the conductivity of photosensitive Si is 300,000 S/m, it is in a metallic state, and photosensitive Ge is in an insulating state, the working state of the metasurface will be switched to a transmission type, and the cross-polarization transmission coefficient in the range of 0.44 THz–1.30 THz is greater than 0.8, and other reflection coefficients and transmission coefficients are all less than 0.3. Similarly, according to the transmission polarization conversion formula, the PCR_t_ in this frequency band reaches 90%, and the relative bandwidth is 98.9%, as shown in Figure 3. Therefore, that design metasurface can realize the reflective and transmissive broadband polarization conversion mode according to different pump light excitation.

**Figure 4:**
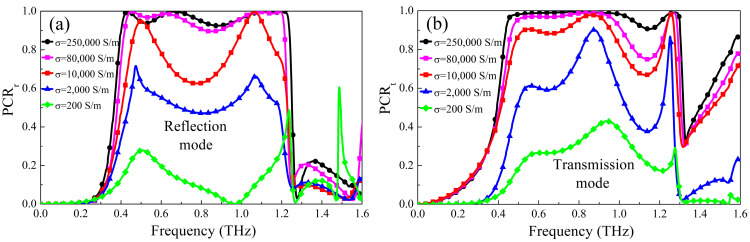
The PCR under different intensities of bump light (**a**) Reflection mode; (**b**) Transmission mode.

Furthermore, adjusting the polarization conversion by the intensity of pump light with different wavelengths is studied. When the designed metasurface works in the reflective mode, as the pump light intensity of 1550 nm wavelength gradually decreases, the metallic conductivity of photosensitive Ge gradually decreases, and the nonmetal gradually increases, so the reflective polarization conversion performance can be gradually tuned, as shown in Figure 4a. As seen from the figure, when the conductivity of Ge decreases to 80,000 S/m, the PCR can still keep about 90%. As the conductivity gradually decreases, the PCR gradually decreases to about 20%. At this time, most electromagnetic waves will be transmitted in the same polarization mode as the incident waves. Similarly, when the metasurface works in the transmission mode, the transmission PCR will be gradually reduced to below 40% as the pump light with the wavelength of 800 nm becomes weaker and weaker. At this time, most electromagnetic waves will be reflected and transmitted in the same polarization mode as the incident waves. Therefore, the designed optically controlled polarization conversion metasurface can control the working efficiency of two polarization conversion modes by the intensity of pump light with different wavelengths. In addition, we compare other recently published multifunctional polarization conversion metasurfaces, as shown in Table 1. The results show that the metasurfaces proposed in this paper not only have reflective and transmissive working modes but also have broadband working bandwidths in the same frequency band, and have the advantage of a simple control mode (RB is relative bandwidth).

### 3.2. Polarization Conversion Mechanism

**Figure 5:**
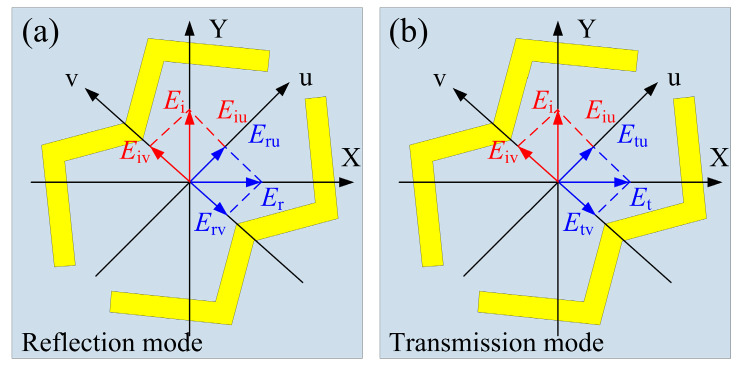
Schematic illustration of the conversion characteristics (**a**) Reflection mode; (**b**) Transmission mode.

The working principle of the designed metasurface can be analyzed by the orthogonal eigenmodes shown in Figure 5. The electromagnetic wave polarized along the Y-axis can be decomposed into two eigenmodes along the u-axis and the v-axis, which are crucial for polarization conversion. When the electromagnetic wave polarized along the Y-axis is incident on the metasurface, its incident, reflected, and transmitted electric fields can be expressed as follows: ${\vec{E}}_{i}=\hat{u}E_{iu}e^{j\phi }+\hat{v}E_{iv}e^{j\phi }$, ${\vec{E}}_{r}=\hat{u}r_{u}E_{iu}e^{j\left(\phi +\phi_{u}\right)}+\hat{v}r_{v}E_{iv}e^{j\left(\phi +\phi_{v}\right)}$, and ${\vec{E}}_{t}=\hat{u}t_{u}E_{iu}e^{j\left(\phi +\phi_{u}\right)}+\hat{v}t_{v}E_{iv}e^{j\left(\phi +\phi_{v}\right)}$, *E_iu_* and *E_iv_* are the incident electric fields along the u -axis and v-axis, *r_u_*(*r_v_*) and *t_u_*(*t_v_*) are the reflection and transmission amplitudes along the u-axis (v-axis), and *φ_u_*(*φ_v_*) is the phase along the u-axis (v-axis). Because of the anisotropy of the designed W-shaped polarization converter, there is a phase difference Δ*φ_vu_* between *φ_u_* and *φ_v_*. For the designed metasurface working in reflection mode, when the conditions of *r_u_* ≈ *r_v_* and Δ*φ_vu_* ≈ 180° are met at the same time, the *E_rv_* will be reversed, and the electric field polarized along the Y-axis will be deflected to the X-axis direction, thus realizing linear polarization conversion. Similarly, when the metasurface works in the transmission mode, if *t_u_* ≈ *t_v_* and Δ*φ_vu_* ≈ 180° are simultaneously satisfied, the transmission polarization conversion performance will be realized.

**Figure 6:**
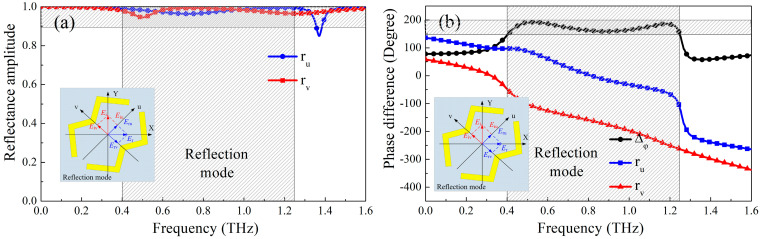
(**a**) Amplitudes and (**b**) phase of polarization conversion mode when the electric field of incident EM waves along the u-axis and v-axis for reflection mode, respectively.

**Figure 7:**
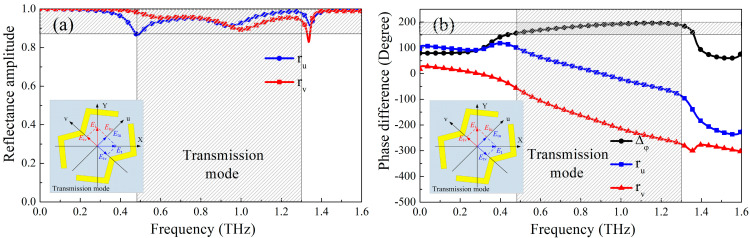
(**a**) Amplitudes and (**b**) phase of polarization conversion mode when the electric field of incident EM waves along the u-axis and v-axis for transmission mode, respectively.

Figure 6 and Figure 7 show the amplitude and phase of the metasurface when the polarization direction of the electric field of the incident electromagnetic wave is along the u-axis and the v-axis. It can be seen from Figure 6a,b that when the metasurface works in the reflective working mode, *r_u_* and *r_v_* are approximately equal in the frequency range of 0.40–1.24 THz, and the phase difference Δ*φ_vu_* is approximately equal to 180°, indicating that the electromagnetic wave polarized along the Y-axis direction incident on the metasurface in this frequency range is converted into the reflected electromagnetic wave polarized along the X-axis direction. Similarly, when the metasurface works in the transmission mode, *t_u_* and *t_v_* are approximate, and Δ*φ_vu_* is close to 180° in the frequency range of 0.44–1.30 THz, as shown in Figure 7a,b, suggesting that the electromagnetic wave polarized along the Y-axis direction is converted into the electromagnetic transmission wave polarized in the X-axis direction.

**Figure 8:**
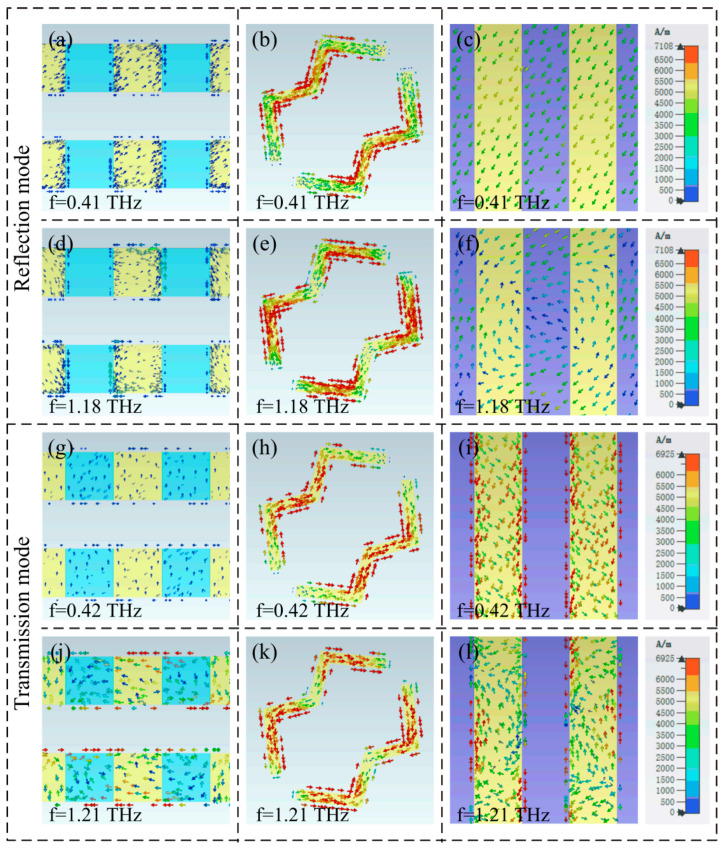
Surface current distribution of (**a**–**f**) Reflection mode; (**g**–**l**) Transmission mode.

To expound on the working mechanism of the designed metasurface, Figure 8 depicts the surface current distribution at the resonance frequency of the metasurface working in reflective and transmissive modes. As can be seen from Figure 8a–f, when the metasurface works in the reflective polarization conversion mode, the surface current on the top metal line is weak, which indicates that the top metal line and semiconductor photosensitive Si are hardly generated in this working mode. The surface current is mainly distributed on the W-shaped resonator and the back plate of the bottom layer, which will excite the magnetic dipole M, which will excite an induced magnetic field H. Because the component H_y_ of the induced magnetic field H along the Y-axis direction is parallel to the electric field component E_i_ of the incident electromagnetic wave, there will be cross-coupling between Hy and E_i_, realizing the polarization conversion from co-polarized wave to cross-polarized wave [9]. In addition, by comparing Figure 8b,e, it is found that the surface current at 0.41 THz of the metasurface is distributed in the middle corners of the W-shaped resonator. In comparison, the current distribution at 1.18 THz can be seen as being divided into two parts, which are located on two arms on both sides of the W-shaped resonator; it is similar to multimode high-order resonance. At this time, the equivalent length of the surface current flowing through is short, so the operating frequency band is high. When the metasurface works in the transmission mode, the surface current at 0.42 THz is distributed on the metal wires of the W-shaped resonator and the back plate, which produces magnetic resonance similar to that at the low frequency of the reflection mode. However, the surface current at 1.21 THz is distributed on the top, middle, and bottom metal lines, which indicates that this resonance position is generated by the mixed resonance mode of three patterned layers, thus realizing the broadband polarization conversion effect.

### 3.3. Analysis of Geometrical Parameters

**Figure 9:**
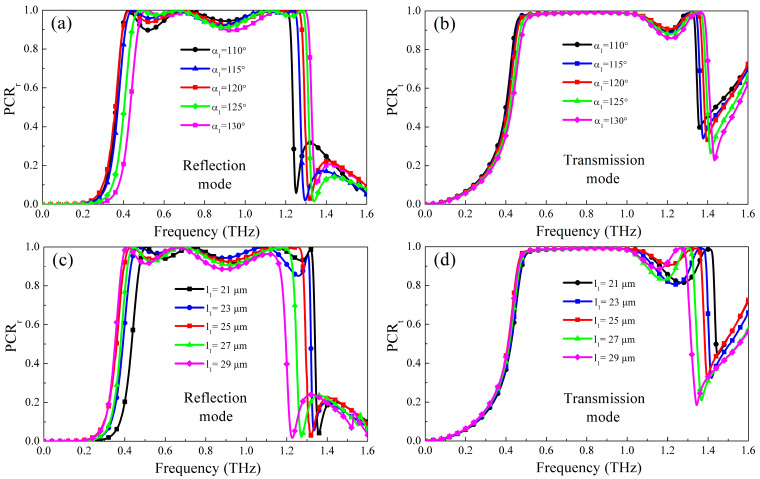
The PCR under different structure parameters (**a**,**b**) different α_1_; (**c**,**d**) l_1_.

**Figure 10:**
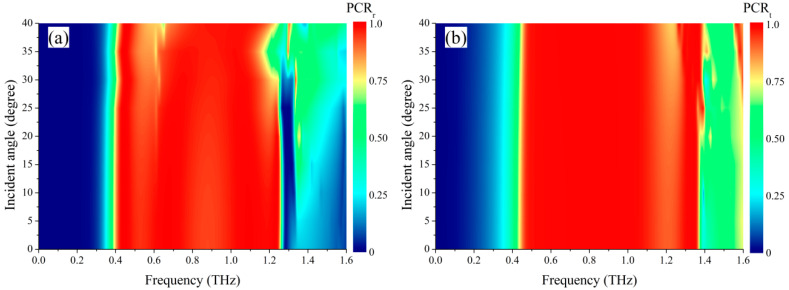
The PCR under different incident angles (**a**) Reflection mode; (**b**) Transmission mode.

When metasurface is applied to the practical environment, the influence of structural parameters on performance should be considered. Figure 9 shows the performance of the designed optically-controlled multifunctional metasurface with different structural parameters *α*_1_ and *l*_1_. Figure 9a,b shows the influence of parameter α_1_ on the working performance of the metasurface. It can be seen from the figure that when working in reflection mode, while keeping other parameters unchanged, the working frequency band gradually moves to the high-frequency band with the gradual increase. This is because when α_1_ increases, the capacitance effect at the corner of the W-shaped resonator weakens, and when the equivalent current flowing length is almost constant, the reduction of the equivalent capacitance will cause the resonance frequency to rise. When the metasurface works in transmission mode, the influence and principle of α_1_ change are similar to reflection mode. Figure 9c,d shows the influence of parameter l_1_ on the working performance of the metasurface. It can be seen from the figures that when l_1_ gradually increases, the working frequency bands of the two working modes will be red-shifted. This is because with the increase of *l*_1_, the length of the equivalent current will increase, and according to the formula: $w\propto c/\pi /eff\sqrt{\varepsilon r}$ [37], its response frequency will decrease. Generally speaking, the electromagnetic wave cannot be incident perpendicular to the metasurface completely, so the stability of the metasurface to the incident angle is a factor to be considered. Figure 10 shows the performance of two polarization conversion working modes of the metasurface at different oblique incident angles. It can be seen from the figure that when the incident angle is within 40°, the PCR and absorption rate can be kept at about 90%, which is better than the report works [19,31]. This profits from the simple pattern and small unit cell of the proposed structure, so the designed metasurface has certain angular stability.

## 4. Conclusions

In this study, a multilayer metasurface with a photosensitive semiconductor embedded in the resonator is designed. According to the electromagnetic simulation analysis, it is concluded that using pump light with different wavelengths to illuminate the metasurface in the terahertz frequency band can realize the switching function between reflective and transmissive modes. And the working efficiency can be tuned by the intensity of the pump light, which has the advantage of simple regulation. In addition, the working principle of the metasurface is analyzed by eigenmode theory and surface current distribution. The stability of the metasurface to structural parameters and incident angle is studied. When the incident angle is 40°, the PCR can be kept at about 90%.

## Figures and Tables

**Table 1:** Comparison with existing polarization converters.

Polarization Converters	Operating Mode	Frequency	RB	Controlled Method
[17]	Transmission	5.72 THz, 13.49 THz, 18.90 THz	-	Voltage-controlled
[22]	Reflection	4.95–9.39 THz	61.9%	Temperature-controlled
[29]	Reflection	11.8–24.1 GHz, 17.7–27.2 GHz	68.5% 42.3%	Voltage-controlled
[33]	Reflection and transmission	0.62–1.70 THz 0.52–1.74 THz,	93.1% 107.9%	Temperature and voltage controlled
This work	Reflection and transmission	0.40–1.24 THz 0.44–1.30 THz	102.4% 98.9%	Optically-controlled
